# Prediction of mortality in secondary peritonitis: a prospective study comparing p-POSSUM, Mannheim Peritonitis Index, and Jabalpur Peritonitis Index

**DOI:** 10.1186/s13741-023-00355-7

**Published:** 2023-12-08

**Authors:** Akshant Anil Pathak, Vivek Agrawal, Naveen Sharma, Karan Kumar, Chinmay Bagla, Aakash Fouzdar

**Affiliations:** 1 Department of General Surgery, University College of Medical Sciences, Delhi, India; 2 Department of General Surgery, AIIMS, Jodhpur, India

**Keywords:** Peritonitis, Outcome, Risk assessment, Prediction

## Abstract

**Background:**

Risk scoring systems are required to allow accurate prognostication, compare outcomes of surgery, and allow patients to make informed decisions about their health. This prospective study compares the p-POSSUM (Portsmouth Modification to Physiological and Operative Severity Score for Enumeration of Mortality), Mannheim Peritonitis Index, and Jabalpur Peritonitis Index for their utility in predicting mortality in patients with peritonitis.

**Methods:**

Perioperative data was collected from 235 patients with secondary peritonitis and used to calculate p-POSSUM, MPI, and JPI scores. The accuracy of the 3 scores was compared using receiver operator characteristic curves.

**Results:**

p-POSSUM and Mannheim Peritonitis Index were similar in their accuracy with area under the curve (AUC) values of 0.756 and 0.757. Jabalpur Peritonitis Index had an AUC of 0.665.

**Conclusion:**

p-POSSUM and Mannheim Peritonitis Index can be used to predict mortality in patients with secondary peritonitis. Jabalpur Peritonitis Index is not suited for this purpose. Further studies are required to improve the diagnostic performance of p-POSSUM and MPI in patients with secondary peritonitis.

## Introduction

Secondary peritonitis and complicated intra-abdominal infections are common causes of emergency surgical admissions worldwide with significant associated mortality and morbidity (Spalding et al. 2008; Sartelli et al. 2014; Malangoni and Inui 2006; Gupta and Kaushik 2006; Yii and Ng 2002). The mortality rates vary from 12 to 41% (Weledji and Ngowe 2013; Pearse et al. 2012), and the primary treatment in most cases requires source control with surgical intervention. The disease profile shows great variation between the developed and developing world with older patients affected more in the West (Malangoni and Inui 2006; Pearse et al. 2012; Salamone et al. 2016) and a more diverse distribution across the ages in developing countries (Gupta and Kaushik 2006; Teleanu et al. 2014; Bali et al. 2014).

This demographic difference may arise due to the difference in aetiologies with chronic diseases and neoplasms found more in the West (Malangoni and Inui 2006). In India, small bowel perforations form a large portion of the cases of peritonitis, with tubercular and enteric perforations contributing significantly (Bali et al. 2014; Ghosh et al. 2016; Jhobta et al. 2006) along with smaller numbers from the causes prevalent in the West. In the setting of peritonitis factors such as the degree of peritoneal contamination, time from onset to operative intervention, cause of peritonitis and source of contamination have been found to significantly alter disease process and outcomes (Tolonen et al. 2019a; Tolonen et al. 2019b).

The patients who require emergency interventions for perforation peritonitis are markedly heterogeneous in their capacity to tolerate surgery and the required postoperative course. Furthermore, many of the underlying surgical pathologies are not readily diagnosed or apparent prior to surgery, especially surgery in an emergency setting (Tolonen et al. 2018). For a prognostic score to be useful in the setting of peritonitis it has to be both accurate and broadly applicable across the range of patient status, intra-abdominal pathology, and the required surgical interventions.

Because of this variation in prognostic factors and outcomes, risk-scoring systems which can predict outcomes and determine prognosis can help the surgeons in making objective decisions during treatment (Sartelli 2010; Koperna 2001). Risk assessment and stratification of patients can also help in surgical audits and assessing quality of care by providing information about expected outcomes which can be then compared to observed outcomes (Deans Gordon and Roy Douglas 1988). They can also help the patients in making more informed decisions regarding procedures and prognosis for a condition that carries high mortality and significant morbidity rates (Anaya and Nathens 2003). While there are multiple scoring systems available to assess mortality risk in surgical patients, their usage is commonly limited by paucity of time and resources. This is especially pertinent in low-resource settings where a highly accurate scoring system may not be useable due to an inability to collate all the required parameters. Another factor to be considered is that some systems require data collected longitudinally over a time period to determine prognosis (Koperna 2001; Fuchs et al. 2019) when the need of the hour may be patient factors at a single point of time to aid surgical decision making. Apart from these, many scoring systems do not take surgical findings and intraabdominal pathology into account even though these contribute significantly to the overall prognosis and risk for a given patient (Sartelli et al. 2014; Tolonen et al. 2019b).

Keeping the above factors in mind, this study was conducted to evaluate the performance in real-world clinical situations of 3 mortality risk scoring systems which are used commonly for prognosticating patients and determining the optimum course of treatment based on easily accessible parameters, routine preoperative investigations, and intraoperative findings, namely the p-POSSUM (Portsmouth Modification to Physiological and Operative Severity Score for Enumeration of Mortality) (Prytherch et al. 1998), MPI (Mannheim Peritonitis Index) (Linder et al. 1987), and Jabalpur Peritonitis Index (JPI) (Mishra et al. 2003).

## Materials and methods

### Ethical statement

The study did not aim to change or modify existing clinical or laboratory practices. Steps were taken to ensure data collection was anonymous. The data collected was not used for making clinical decisions.

This study was reviewed and cleared by the Institutional ethics committee prior to the study as it was carried out as a thesis project for post-graduate degree certification.

### Study design and setting

The study was a prospective observational cohort study carried out at the Department of Surgery, University College of Medical Sciences, and GTB Hospital, Delhi between December 2018 and March 2020. The hospital is a 1800-bedded multi-specialty tertiary care facility with facilities for acute and emergency surgery and a dedicated surgical ICU. Approximately 600–800 surgeries for gastrointestinal emergencies are carried out per year with the bulk being made up of secondary peritonitis followed by bowel obstruction.

The intention behind the study was to evaluate three different risk scoring systems in current use by surgical teams at the institution and affiliated hospitals for the purposes of triage, resource allocation, and prognostication.

The study has been written in conformation with STROBE Guidelines for cohort studies.

### Inclusion and exclusion criteria

All consecutive patients of non-traumatic secondary peritonitis undergoing emergency laparotomy by three different general surgical units in this period were included in the study. Secondary Peritonitis was defined as an intra-abdominal infection that extended beyond the organ of origin and caused either a localized or diffuse inflammation of the peritoneum with soiling of the peritoneal cavity.

The exclusions were as follows:

- Patients with traumatic perforations due to blunt or penetrating trauma.
- Postoperative peritonitis due to leaks who had undergone index surgery elsewhere.
- Those who could not be taken up for surgery either due to lack of consent or preoperative death were excluded from the study.

### Outcome measures

The primary outcome measure was postoperative mortality (either in a hospital or within 90 days of the procedure if discharged) or survival.

### Treatment

Every patient followed the same standard pathway using the Surviving Sepsis Guidelines (Rhodes et al. 2016). Clinical and biochemical assessment was carried out to determine and classify the presence of sepsis, septic shock, and organ dysfunction according to internationally accepted criteria (Sartelli et al. 2014; Sartelli et al. 2012; Rhodes et al. 2017). After confirmation of diagnosis and adequate resuscitation, patients were taken up for exploratory laparotomy after pre-anesthetic assessment by the anesthesia team on duty. The procedure performed was decided by the operating surgeon, either the consultant on duty or senior resident after a discussion with the consultants.

### Data collection

The clinical findings were recorded from hospital preoperative notes, operative notes, anesthetic charts, and postoperative ward notes. After the initial registry, patients were followed till the end of their stay in the hospital (discharge or mortality). For patients who were discharged to home, follow-up visits occurred at 7 days, 28 days, and 3 months.

The data collected was of the following types:

1. Preoperative data including demographic data, co-morbid history, examination findings, laboratory investigations, and radiological findings.
2. Intraoperative findings, i.e., degree of contamination, etiology of perforation, source of contamination, intraoperative blood loss, method of abdomen closure, and need for blood transfusion.
3. Postoperative course including the need for ICU stay, course of disease, and any postoperative complications.

The final etiology was defined by intraoperative findings, histopathological, and microbiological examination.

Postoperative mortality was defined as intrahospital death or death within 90 days of the index procedure.

To reduce bias, all consecutive patients with secondary peritonitis who underwent laparotomy were included in the study. All the data points required for the calculation of scores were collected from patient records and verified by two different investigators.

### Statistical analysis and scoring systems

The scoring systems to be evaluated were chosen after taking a survey of multiple surgeons at our institution and affiliated hospitals about the risk scoring systems being used commonly to prognosticate and triage patients. The scoring systems used were p-POSSUM (Portsmouth Modification to Physiological and Operative Severity Score for Enumeration of Mortality) (Prytherch et al. 1998), MPI (Mannheim Peritonitis Index) (Linder et al. 1987), and Jabalpur Peritonitis Index (JPI) (Mishra et al. 2003).

Using the patient data and variables, risk scores for every patient, under each of the three systems to be assessed were calculated. Receiver operator characteristic (ROC) curves were constructed for sensitivity analysis for each of the 3 risk-scoring systems. These ROCs are used to determine diagnostic performance and compare the three scores based on the area under the curve (AUC) (Soreide 2009). The receiver operator characteristic curve was also used to define a cutoff score, using the Youden Index (Safari et al. 2016) beyond which patients were considered to be high risk (Safari et al. 2016). Based on the stratification of patients into high- or low-risk populations and the mortality rate in these, sensitivity, specificity, positive predictive value, and negative predictive value were calculated.

After cutoff scores were calculated using ROC, further calibration of scores was done using chi-square test for observed to expected mortality rates (Oliver et.al 2015) to ensure the applicability of results.

*p* value of < 0.05 was considered significant.

### Scoring systems

Both p-POSSUM and MPI are commonly used systems that have been reported to have high accuracy based on the area under the curve (AUROC) in receiver operator characteristic curves (González-Pérez et al. 2019; Neary et al. 2007; Scott et al. 2014) with AUROC greater than 80% indicating good diagnostic ability (Safari et al. 2016; Hanczar et al. 2010). The Jabalpur peritonitis index is easy to use with few components and perhaps more suited to Indian populations as the original patient cohort was based in India. Due to its simplicity and low number of variables, it is also commonly used in low-resource settings where extensive preoperative workup may not always be feasible.

### p-POSSUM

p-POSSUM, standing for Portsmouth Modification to Physiological and Operative Severity Score for the Enumeration of Mortality was devised by Prytherch et al. (1998). The system uses a 12-factor physiological score for patient condition prior to surgery and a 6-factor operative severity score, both of which were derived from earlier observations on 1372 patients (Copeland et al. 1991). The physiological and operative scores are used to give a predicted percentage risk of mortality for a patient by calculating via the p-POSSUM equation as follows:

$$\mathrm{ln }[\mathrm{R }/ (1-\mathrm{R})]\hspace{0.17em}=-9.065+(0.1692\hspace{0.17em}\times \hspace{0.17em}\mathrm{physiological\, score})\hspace{0.17em}+\hspace{0.17em}(0.1550\hspace{0.17em}\times \hspace{0.17em}\mathrm{operative \,severity \,score})$$

where *R* is the predicted risk of mortality.

### Mannheim Peritonitis Index (MPI)

Based on the clinical observations and risk factors from 1243 patients of purulent peritonitis, Linder et al. (1987) devised the Mannheim Peritonitis Index for predicting mortality in patients of perforation peritonitis. A total of 8 factors are included in the scoring system covering demographic, physiological, and disease-specific factors. The total score possible is 47. In the original study, with a cutoff value of > 26, MPI helped in identifying patients at increased risk of mortality with good sensitivity ( 84%), specificity (79%), and overall accuracy (81%).

### Jabalpur Peritonitis Index

Mishra et al. (2003) devised the Jabalpur peritonitis index for perforation peritonitis as a simplified system for use in resource-poor situations where extensive preoperative investigations may not be available. One hundred forty patients were studied prospectively, and multiple regression analysis was employed to identify 6 factors which had a high association with mortality. Using 9 as a cutoff value, beyond which 50% mortality was observed, the authors determined the system to have a sensitivity of 87% and specificity of 85%.

## Results

### Patient characteristics

A total of 235 patients of secondary, non-traumatic, bacterial peritonitis who underwent emergency laparotomy with a mean age of 36.58 years (range 11—85 years) were included in the current study. The largest number of patients (*n* = 113) were in the age group 18–40 years. Eighty patients were females (34.04%), and 155 were males (65.9%) (Table 1). One hundred nineteen out of 235 patients had organ dysfunction at the time of admission as defined by biochemical and physical parameters. The onset to intervention time was measured from the time of onset of symptoms to the time of the start of surgery. The admission to operation interval was measured from the time of admission to the time of the start of surgery (Table 2).

**Table 1:** Patient demographics

	**Parameter**	**Survived**	**Died**
**Gender**	MALE	122 (78.7%)	33 (21.3%)
	FEMALE	48 (60.0%)	32 (40.0%)
	TOTAL	170 (72.3%)	65 (27.7%)
**Age**	< 18 Years	16 (66.7%)	8 (33.3%)
	18–40 Years	87 (77.0%)	26 (23.0%)
	40–60 Years	42 (68.9%)	19 (31.1%)
	> 60 Years	25 (67.6%)	12 (32.4%)
	Total	170 (72.3%)	65 (27.7%)
**Mean age**		35.68 years	38.95 years
**Number of known comorbidities**	None	104 (83.2%)	21 (16.8%)
	1	37 (78.7%)	10 (21.2%)
	2	19(52.7%)	17 (47.2%)
	3	9 (37.5%)	15 (62.5%)
	** ≥ 4**	**1 (33.3%)**	**2 (66.7%)**

**Table 2:** Preoperative findings

		**Number of patients**	**Survived**	**Died**
Duration of disease (onset to intervention)	< 1 Day	16	13 (81.25%)	3 (18.75%)
	1–3 Days	129	100 (77.5%)	29 (22.5%)
	3–5 Days	54	35 (64.8%)	19 (35.2%)
	> 5 Days	36	22 (61.1%)	14 (38.8%)
Admission to operation interval (hours)	0–6 H	36	26 (72.2%)	10 (27.8%)
	6–12 H	111	89 (80.2%)	22 (19.8%)
	12–24 H	67	39 (58.2%)	28 (41.8%)
	> 24 H	21	16 (76.2%)	5 (23.8%)
Organ dysfunction	Yes	119	61 (51.3%)	58 (48.7%)
	No	116	109 (93.9%)	7 (6.03%)
Number of organ systems affected preoperatively	None	116	109 (93.9%)	7 (6.1%)
	1 System	58	37 (63.8%)	21 (36.2%)
	2 Systems	36	16 (44.4%)	20(55.5%)
	3 Systems	22	8 (36.3%)	14 (63.6%)
	4 Systems	3	0 (0.0%)	3 (100.0%)

### Intraoperative findings

#### Extent of contamination

Two hundred eleven out of 235 patients had diffuse peritonitis with multi-quadrant contamination intraoperatively. Twenty-four patients had localized peritonitis with contamination only in a single quadrant (Table 3).

**Table 3:** Intraoperative findings

		**Number of patients**	**Survived**	**Died**
Extent of contamination	Localized	24	22 (91.7%)	2 (8.3%)
	Diffuse	211	148 (70.1%)	63 (29.9%)
**Source of Contamination**	Gastro-Duodenal	54	41 (75.9%)	13 (24.1%)
	Jejunal	25	9 (36.0%)	16 (64.0%)
	Ileal	80	65 (81.2%)	15 (18.8%)
	Colon	32	19 (59.4%)	13 (40.6%)
	Appendix	17	17 (100.0%)	0 (0.0%)
	Recto-Sigmoid	14	10 (71.4%)	4 (28.6%)
	Hepato-biliary	6	5(83.3%)	1(16.6%)
	Others	1	1 (100.0%)	0 (0.0%)
	Not Found	6	3 (50.0%)	3 (50.0%)
**Etiology**	Amoebic	15	8 (53.3%)	7 (46.7%)
	Appendicular Gangrene/Perforation	17	17 (100.0%)	0 (0.0%)
	Bowel Ischemia	11	8 (72.7%)	3 (27.3%)
	Chronic peptic ulcer	47	36 (76.6%)	11 (23.4%)
	Enteric	22	18 (81.8%)	4 (18.2%)
	Liver Abscess rupture	3	3 (100.0%)	0 (0.0%)
	Neoplasm	22	15 (68.2%)	7 (31.8%)
	Obstruction	5	4 (80.0%)	1 (20.0%)
	Tubercular	69	46 (66.7%)	23 (33.3%)
	Others	24	15 (62.5%)	9 (37.5%)

### Source of contamination and etiology

Of the 235 patients included in the study, the source could not be localized in 6 patients due to dense intraperitoneal adhesions, omental thickenings, and sclerosing peritonitis.

Overall, the most common site of perforation was the ileum (*n* = 80; 34%) followed by gastro-duodenal perforations (*n* = 54; 23%).

The most common etiology for perforation was tubercular enteritis and tubercular peritonitis (69 out of 235) followed by gastroduodenal ulceration (47 out of 235).

Twenty-two out of 235 patients had a perforation secondary to a neoplasm, either detected at the time of surgery or on histopathological analysis of the resected specimen (Table 3).

### Outcomes and postoperative course

Out of 235 patients, there were a total of 65 deaths for an overall mortality rate of 27.7%. The maximum number of mortalities was in the time period ≤ 7 days from surgery (36 out of 65). Thirty-two of the 36 patients who died in this time period had organ dysfunction and severe sepsis prior to surgery.

### Scoring systems

#### p-POSSUM

The area under the receiver operator characteristic curve (AUROC) for p-POSSUM in predicting the outcome, died or survived was 0.756 (95% CI 0.688–0.823), demonstrating fair diagnostic performance. It was statistically significant (*p* =  < 0.001). Based on the receiver operator characteristic curve, the ideal cutoff score for p-POSSUM in predicting mortality was 29.1%. At this cutoff score of 29.1%, p-POSSUM predicted mortality with a sensitivity of 88% (95% CI 77–95) and a specificity of 52% (95% CI 44–59).

**Fig. 1:**
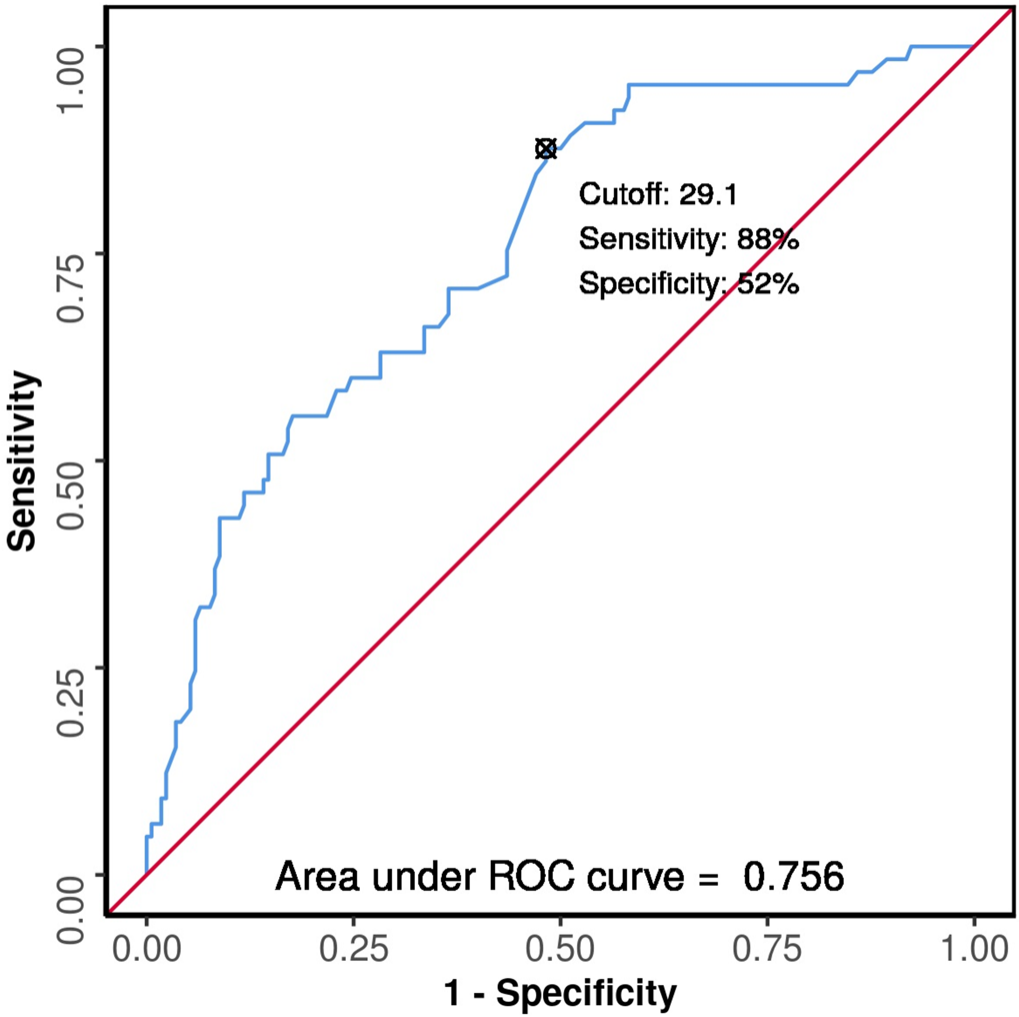
ROC curve for p-POSSUM

The odds ratio (95% CI) of mortality in patients with p-POSSUM score ≥ 29.1% was 6.68 (95% CI 3.11–14.36) as compared to those patients who had scores below the cutoff score (Fig. 1).

### Mannheim Peritonitis Index

The area under the ROC curve (AUROC) for MPI in predicting mortality in cases of perforation peritonitis was 0.757 (95% CI 0.691–0.824), which is considered as fair diagnostic performance. It was statistically significant (*p* =  < 0.001). Based on the ROC, the ideal cutoff value for the Mannheim Peritonitis Index in predicting mortality was 27. At a cutoff of MPI ≥ 27, MPI predicted mortality with a sensitivity of 85% (95% CI 74–92) and a specificity of 58% (95% CI 50–65).

**Fig. 2:**
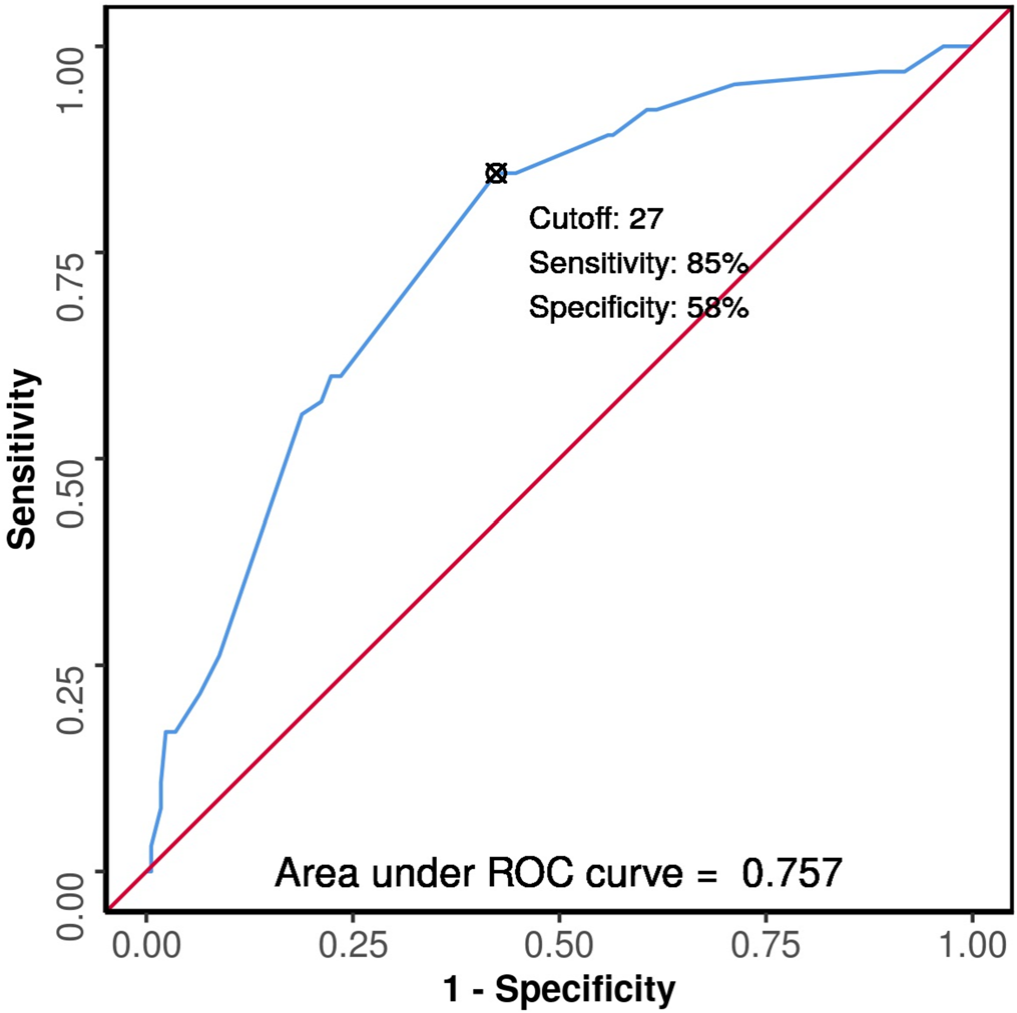
Receiver operator characteristic curve for MPI

The odds ratio (95% CI) of mortality in patients with MPI score ≥ 27 was 4.88 (95% CI 2.65–8.97) as compared to those patients who had scores below the cutoff (Fig. 2).

### Jabalpur Peritonitis Index

**Fig. 3:**
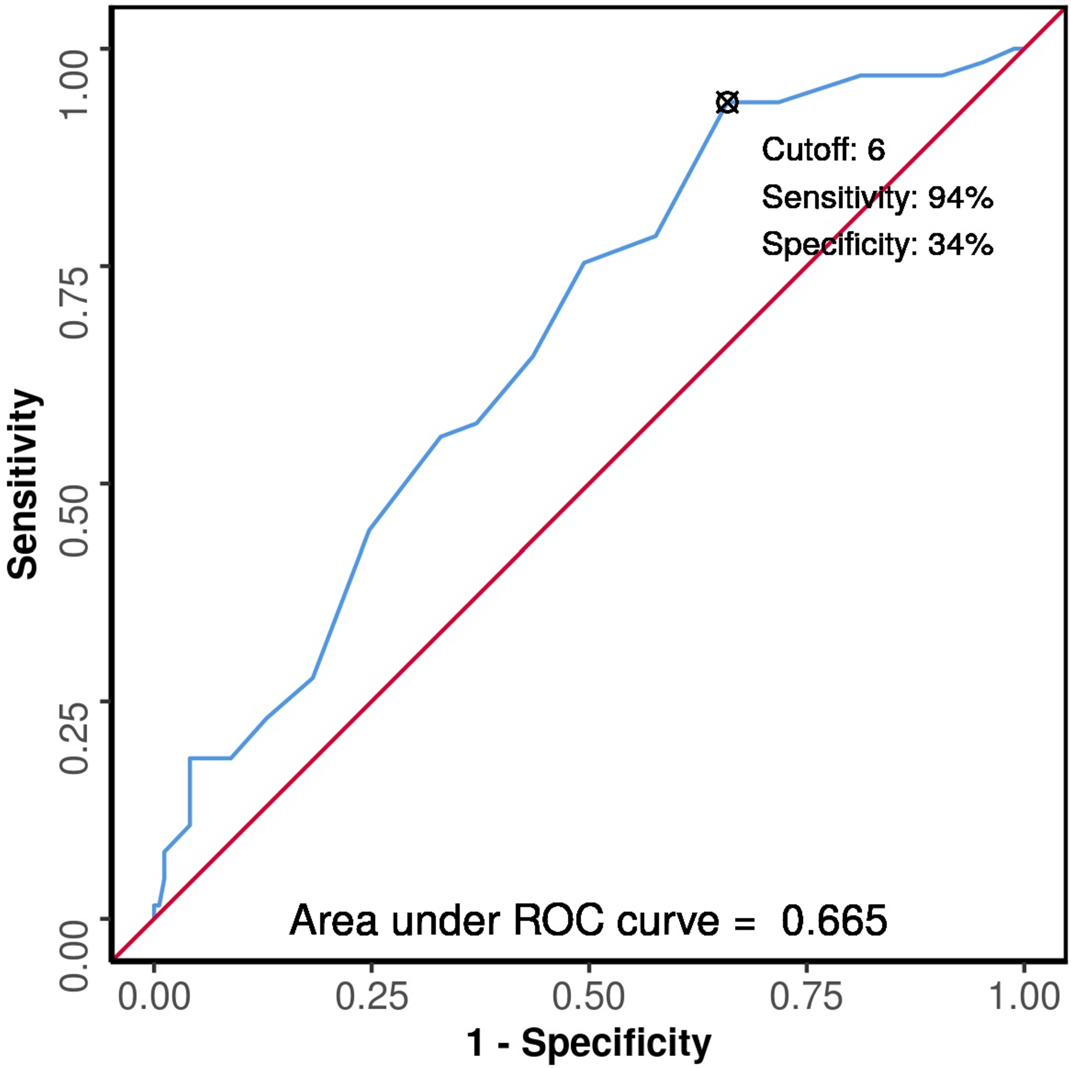
Receiver operator characteristic for JPI

The area under the ROC curve (AUROC) for JPI in predicting mortality versus survival was 0.665 (95% CI 0.592–0.739), thus demonstrating poor diagnostic performance. Based on the receiver operator characteristic curve, the ideal cutoff value for dividing patients into high-risk and low-risk groups was 6. At a cutoff score of ≥ 6, the Jabalpur Peritonitis Index predicted mortality with a sensitivity of 94% (95% CI 85–98) and a specificity of 34% (95% CI 27–42) (Fig. 3).

The odds ratio (95% CI) of mortality in patients with JPI score ≥ 6 was 2.68 (95% CI 1.38–5.2) as compared to those patients who had scores below the cutoff.

Comparing the 3 scoring systems (Table 4), p-POSSUM and Mannheim Peritonitis Index had almost equivalent diagnostic performance, while Jabalpur Peritonitis Index had poor diagnostic performance. While all three scores had good sensitivity, they had low specificity in predicting mortality. All three of the scores had very high negative predictive value, indicating their utility in identifying low-risk cases more than high-risk cases.

**Table 4:** Comparison of scores

Score	Cutoff score	Sensitivity	Specificity	PPV	NPV	Area under curve	Chi-square value
p-POSSUM	29.1%	88%	52%	41%	91.7%	0.756	30.2965
MPI	27	85%	58%	43.3%	90.7%	0.757	33.8152
JPI	6	94%	34%	35.3%	93.5%	0.665	18.9315

There was a significant correlation between being in the high-risk group (Table 5) in any of the 3 scores and mortality (*p* < 0.01) on calibration using chi-square test. On division into high-risk and low-risk groups based on cutoff scores, the patients in high-risk groups under each of the 3 systems had a significantly higher risk of mortality as shown by the odds ratio for mortality earlier.

**Table 5:** Comparison of scores in high-risk and low-risk patients

Score	Cutoff score	Total positives (high risk)	True positives (died)	True negatives (low risk)	False positives (high risk but survived))	False negatives (low risk but died)
p-POSSUM	29.1%	139 (59.1%)	57 (24%)	88 (37%)	82 (35%)	8 (3%)
MPI	27	127 (54.0%)	55 (23%)	98 (42%)	72 (31%)	10 (4%)
JPI	6	173 (73.6%)	61 (26%)	58 (25%)	112 (48%)	4 (2%)

## Discussion

The objective of our study was to compare the performance of 3 scoring systems, p-POSSUM, Mannheim peritonitis Index, and Jabalpur peritonitis index in predicting mortality in patients with perforation peritonitis using receiver operator characteristic curves.

Of the three scores under evaluation, Jabalpur Peritonitis index had a poor diagnostic performance with an area under the curve (AUC) of 0.665, whereas Mannheim Peritonitis Index and p-POSSUM had almost equivalent performance with AUC of 0.757 and 0.756, respectively, which can be considered as fair diagnostic performance (Safari et al. 2016; Oliver et al. 2015). Based on the overall fair diagnostic performance as assessed by receiver operator characteristic curve (Oliver et al. 2015; Hanczar et al. 2010), comparable performance with other studies and independent correlation with mortality, p-POSSUM and Mannheim Peritonitis Index can be used for risk assessment in perforation peritonitis. However, these scores also require updating and re-evaluation as the performance is on the lower side as compared to other scores (Sartelli et al. 2015).

The performance of the systems was also worse than that reported in many other studies as many studies have reported AUC for p-POSSUM to be above 90% (Hobson et al. 2007; Nachiappan and Litake 2016; Mohil et al. 2004). Similarly, the reported AUC for the Mannheim Peritonitis Index in predicting mortality is above 0.8 (González-Pérez et al. 2019; Mineccia et al. 2016). This difference may arise from the fact that we had a significantly higher portion of patients with organ dysfunction at the time of admission in our study and the scores do not include all the parameters that define organ dysfunction (Dellinger et al. 2013), thereby underestimating the risk of mortality in these patients as patients with significant organ dysfunction may still not be given a higher risk score because they fall outside the parameters of the scoring system.

There is also the fact that most of the studies reporting good performance of the scoring systems have smaller sample sizes and a higher proportion of low-risk patients (Mohil et al. 2004; Yelamanchi et al. 2020). This is also seen in Indian studies where despite the high incidence of disease, few patients are included in studies resulting in small sample sizes (Gupta and Kaushik 2006; Chakma et al. 2013). As has been reported in many studies, p-POSSUM specifically overestimates mortality in low-risk cases and slightly underestimates mortality in high-risk cases (Neary et al. 2007; Mohil et al. 2004).

The cutoff scores for the different systems beyond which a patient was to be deemed high risk in our study differed from those described by the original authors (Linder et al. 1987; Mishra et al. 2003).

Of the three systems under evaluation, p-POSSUM may be best suited for usage in clinical audits as the patients can be grouped in risk strata and observed to expected outcomes derived easily.

Mortality rates for perforation peritonitis range from 10 to 33% (Salamone et al. 2016; Anaya and Nathens 2003). The mortality rate in our study was 27.7% (*n* = 65). As compared to most other studies, we had a very high portion of patients with sepsis and organ dysfunction, which strongly correlates with mortality in peritonitis (Sartelli et al. 2014; Anaya and Nathens 2003; Pacelli 1996).

The age distribution in our study was similar to other studies conducted in Indian populations (Bali et al. 2014; Jhobta et al. 2006) but on the lower side compared to Western studies (Anaya and Nathens 2003; Gauzit et al. 2009). A possible cause for this may be the increased incidence of perforation due to infectious causes in Indian populations. All three risk scoring systems in this study utilize age as one of the risk factors involved in determining the risk of mortality, although different age cutoffs are used in each of the systems.

Similar to other studies (Payá-Llorente et al. 2020), there was an increase in mortality rates with an increasing number of prior comorbid conditions. Of the three risk-scoring systems assessed in this study, only the p-POSSUM takes patient comorbidities into consideration (Prytherch et al. 1998) while the Mannheim and Jabalpur peritonitis indices make no separate provision for concurrent illness.

Multiple studies have found that the duration of symptoms beyond 24 h significantly worsens mortality (Sartelli et al. 2014; Sartelli et al. 2015) and morbidity (Payá-Llorente et al. 2020). In our study, only 16 patients presented within 24 h of symptom onset and this subgroup had a lower mortality rate than patients who presented beyond 24 h of disease onset. Both the Mannheim and Jabalpur Index ascribe points for delayed presentation while calculating scores for patients; however, this important factor is not included in p-POSSUM.

Of the 3 scoring systems, only the MPI gives risk points based on the source of contamination, a factor found to have a significant correlation with poor outcomes (Sartelli et al. 2014) and mortality rates (Sartelli et al. 2015). The difference in mortality rates based on the source of contamination was also observed in our study.

To conclude, all 3 risk-scoring systems have clinical utility in predicting the risk of mortality in patients with perforation peritonitis; however, there are distinct shortcomings in the systems and upgradation of parameters to more recent definitions may be required to increase accuracy.

### Limitations

The study was a single-center study. As a tertiary care center, the majority of patients included in the study were significantly sicker with more organ impairment and higher risk as compared to the general population at most other centers. Some factors such as duration of symptoms cannot be quantified with complete accuracy as they are subjective and based on patient recall rather than an objective measure Despite a larger sample size than older reported studies, the sample size may still be smaller than that required to generate findings applicable to the general population.

## Conclusions

p-POSSUM and Mannheim Peritonitis Index can be cautiously used in assessing the risk of mortality in patients with perforation peritonitis, while the Jabalpur Peritonitis Index is not suited for this.

The profile of perforation peritonitis differs significantly in developing countries as compared to the Western world, and large, multi-centric cohort studies are needed to accurately describe the risk and prognostic factors that can influence outcomes in these populations. Larger studies are also required to compare the surgical systems currently in use with newer, physiological risk systems.

## Data Availability

Datasets used and analyzed during the study are available on request from the corresponding author.

## References

[CR1] Anaya DA, Nathens AB (2003). Risk factors for severe sepsis in secondary peritonitis. Surg Infect (larchmt).

[CR2] Bali RS, Verma S, Agarwal PN, Singh R, Talwar N. Perforation peritonitis and the developing world. ISRN Surg. 2014;2014:1–4. Available from: http://www.hindawi.com/journals/isrn/2014/105492/10.1155/2014/105492 PMC4004134 25006512

[CR3] Chakma SM, Singh RL, Parmekar MV, Gojen Singh KH, Kapa B, Sharatchandra Singh KH (2013). Spectrum of perforation peritonitis. J Clin Diagnostic Res.

[CR4] Copeland GP, Jones D, Walters M. POSSUM: a scoring system for surgical audit. Br J Surg. 1991;78(3):355–60. Available from: http://www.ncbi.nlm.nih.gov/pubmed/2021856 10.1002/bjs.1800780327 2021856

[CR5] Deans Gordon T, Roy Douglas AOSW (1988). Auditing perioperative mortality. Ann R Coll Surg Engl..

[CR6] Dellinger RP, Levy M, Rhodes A, Annane D, Gerlach H, Opal SM (2013). Surviving sepsis campaign: international guidelines for management of severe sepsis and septic shock, 2012. Intensive Care Med.

[CR7] Fuchs PA, Czech IJ, Krzych ŁJ (2019). The pros and cons of the prediction game: the never-ending debate of mortality in the intensive care unit. Int J Environ Res Public Health..

[CR8] Gauzit R, Péan Y, Barth X, Mistretta F, Lalaude O (2009). Epidemiology, management, and prognosis of secondary non-postoperative peritonitis: a French Prospective observational multicenter study. Surg Infect (larchmt).

[CR9] Ghosh PS, Mukherjee R, Sarkar S, Halder SK, Dhar D. Epidemiology of secondary peritonitis: analysis of 545 cases. Int J Sci Study. 2016;3(12):12. Available from: www.ijss-sn.com

[CR10] González-Pérez LG, Sánchez-Delgado Y, Cruz-Manzano JF, Gutiérrez-Uvalle GE, Gracida-Mancilla NI (2019). Index of Mannheim and mortality in sepsis abdominal. Cirugía y Cir (English Ed.

[CR11] Gupta S, Kaushik R (2006). Peritonitis - the Eastern experience. World J Emerg Surg.

[CR12] Hanczar B, Hua J, Sima C, Weinstein J, Bittner M, Dougherty ER (2010). Small-sample precision of ROC-related estimates. Bioinformatics.

[CR13] Hobson SA, Sutton CD, Garcea G, Thomas WM (2007). Prospective comparison of POSSUM and P-POSSUM with clinical assessment of mortality following emergency surgery. Acta Anaesthesiol Scand.

[CR14] Jhobta RS, Attri AK, Kaushik R, Sharma R, Jhobta A (2006). Spectrum of perforation peritonitis in India-review of 504 consecutive cases. World J Emerg Surg.

[CR15] Koperna T (2001). Risk stratification in emergency surgical patients. Arch Surg.

[CR16] Linder MM, Wacha H, Feldmann U, Wesch G, Streifensand RA, Gundlach E. The Mannheim peritonitis index. An instrument for the intraoperative prognosis of peritonitis. Chirurg. 1987;58(2):84–92. Available from: http://www.ncbi.nlm.nih.gov/pubmed/3568820 3568820

[CR17] Malangoni MA, Inui T (2006). Peritonitis - the Western experience. World J Emerg Surg.

[CR18] Mineccia M, Zimmitti G, Ribero D, Giraldi F, Bertolino F, Brambilla R, et al. Improving results of surgery for fecal peritonitis due to perforated colorectal disease: a single center experience. Int J Surg. 2016;25:91–7. Available from: 10.1016/j.ijsu.2015.11.028 10.1016/j.ijsu.2015.11.028 26639085

[CR19] Mishra A, Sharma D, Raina VK (2003). A simplified prognostic scoring system for peptic ulcer perforation in developing countries. Indian J Gastroenterol..

[CR20] Mohil RS, Bhatnagar D, Bahadur L, Rajneesh, Dev DK, Magan M. POSSUM and P-POSSUM for risk-adjusted audit of patients undergoing emergency laparotomy. Br J Surg. 2004;91(4):500–3. Available from: http://www.ncbi.nlm.nih.gov/pubmed/15048756 10.1002/bjs.4465 15048756

[CR21] Nachiappan M, Litake MM (2016). Scoring systems for outcome prediction of patients with perforation peritonitis. J Clin Diagnostic Res..

[CR22] Neary WD, Prytherch D, Foy C, Heather BP, Earnshaw JJ (2007). Comparison of different methods of risk stratification in urgent and emergency surgery. Br J Surg.

[CR23] Oliver CM, Walker E, Giannaris S, Grocott MPW, Moonesinghe SR (2015). Risk assessment tools validated for patients undergoing emergency laparotomy: a systematic review. Br J Anaesth.

[CR24] Pacelli F. Prognosis in Intra-abdominal Infections. Arch Surg. 1996;131(6):641. Available from: 10.1001/archsurg.1996.01430180067014 10.1001/archsurg.1996.01430180067014 8645072

[CR25] Payá-Llorente C, Martínez-López E, Sebastián-Tomás JC, Santarrufina-Martínez S, de'Angelis N, Martínez-Pérez A (2020). The impact of age and comorbidity on the postoperative outcomes after emergency surgical management of complicated intra-abdominal infections. Sci Rep..

[CR26] Pearse RM, Moreno RP, Bauer P, Pelosi P, Metnitz P, Spies C, et al. Mortality after surgery in Europe: a 7 day cohort study. Lancet. 2012;380(9847):1059–65. Available from: 10.1016/S0140-6736(12)61148-9 10.1016/S0140-6736(12)61148-9 PMC3493988 22998715

[CR27] Prytherch DR, Whiteley MS, Higgins B, Weaver PC, Prout WG, Powell SJ. POSSUM and Portsmouth POSSUM for predicting mortality. Br J Surg. 1998;85(9):1217–20. Available from: http://www.ncbi.nlm.nih.gov/pubmed/9752863 10.1046/j.1365-2168.1998.00840.x 9752863

[CR28] Rhodes A, Evans LE, Alhazzani W, Levy MM, Antonelli M, Ferrer R (2017). Surviving sepsis campaign: international guidelines for management of sepsis and septic shock: 2016. Intensive Care Med.

[CR29] Scott S, Lund JN, Gold S, Elliott R, Vater M, Chakrabarty MP, et al. An evaluation of POSSUM and P-POSSUM scoring in predicting post-operative mortality in a level 1 critical care setting. BMC Anesthesiol. 2014; 18;14(1):104. Available from: http://link.springer.com/10.1007/s00268-012-1683-0 10.1186/1471-2253-14-104 PMC4247634 25469106

[CR30] Safari S, Baratloo A, Elfil M, Negida A (2016). Evidence based emergency medicine; part 5 receiver operating curve and area under the curve. Emerg (Tehran, Iran)..

[CR31] Salamone G, Licari L, Falco N, Augello G, Tutino R (2016). original article Mannheim Peritonitis Index ( MPI ) and elderly population : prognostic. G chic..

[CR32] Spalding DRC, Williamson RCN. Peritonitis. Br J Hosp Med (Lond) [Internet]. 2008 Jan;69(1):M12–5. Available from: http://www.ncbi.nlm.nih.gov/pubmed/18293728 10.12968/hmed.2008.69.Sup1.28050 18293728

[CR33] Sartelli M, Abu-Zidan FM, Catena F, Griffiths EA, Di Saverio S, Coimbra R, et al. Global validation of the WSES Sepsis Severity Score for patients with complicated intra-abdominal infections: a prospective multicentre study (WISS Study). World J Emerg Surg. 2015;10(1):1–8. Available from: 10.1186/s13017-015-0055-0 10.1186/s13017-015-0055-0 PMC4681030 26677396

[CR34] Sartelli M, Catena F, Ansaloni L, Coccolini F, Corbella D, Moore EE (2014). Complicated intra-abdominal infections worldwide: the definitive data of the CIAOW Study. World J Emerg Surg.

[CR35] Sartelli M (2010). A focus on intra-abdominal infections. World J Emerg Surg.

[CR36] Soreide K. Receiver-operating characteristic curve analysis in diagnostic, prognostic and predictive biomarker research. J Clin Pathol. 2009;62(1):1–5. Available from: http://jcp.bmj.com/cgi/doi/10.1136/jcp.2008.061010 10.1136/jcp.2008.061010 18818262

[CR37] Teleanu G, Iordache F, Beuran M (2014). Prognostic scoring systems-validation and their utility in patients with abdominal sepsis in colon peritonitis. J Med Life.

[CR38] Tolonen M, Kuuliala K, Kuuliala A, Leppäniemi A, Kylänpää ML, Sallinen V (2019). The association between intraabdominal view and systemic cytokine response in complicated intraabdominal infections. J Surg Res.

[CR39] Tolonen M, Sallinen V, Leppäniemi A, Bäcklund M, Mentula P (2019). The role of the intra-abdominal view in complicated intra-abdominal infections. World J Emerg Surg.

[CR40] Tolonen M, Coccolini F, Ansaloni L, Sartelli M, Roberts DJ, McKee JL (2018). Getting the invite list right: a discussion of sepsis severity scoring systems in severe complicated intra-abdominal sepsis and randomized trial inclusion criteria. World J Emerg Surg.

[CR41] Weledji EP, Ngowe MN. The challenge of intra-abdominal sepsis. Int J Surg. 2013;11(4):290–5. Available from: 10.1016/j.ijsu.2013.02.021 10.1016/j.ijsu.2013.02.021 23473994

[CR42] Yelamanchi R, Gupta N, Durga CK, Korpal M. Comparative study between P- POSSUM and Apache II scores in predicting outcomes of perforation peritonitis: prospective observational cohort study. Int J Surg. 2020;83(August):3–7. Available from: 10.1016/j.ijsu.2020.09.006 10.1016/j.ijsu.2020.09.006 32927143

[CR43] Yii MK, Ng KJ (2002). Risk-adjusted surgical audit with the POSSUM scoring system in a developing country. Br J Surg.

